# Supplementary material for: Differential Response to Single and Combined Salt and Heat Stresses: Impact on Accumulation of Proteins and Metabolites in Dead Pericarps of Brassica juncea
Source: Int J Mol Sci. 2021 Jun 30;22(13):7076. doi: 10.3390/ijms22137076 (PMC8267682; doi:10.3390/ijms22137076)
Supplement: Supplementary file 1 [file ijms-22-07076-s001.zip › Supplementary Figs S1 & S2.pdf]

Supplementary materials

Differential response to single and combined salt and heat stresses: impact on accumulation of proteins and metabolites in dead pericarps of *Brassica juncea*

Jeevan R. Singiri[1]†, Bupur Swetha[1]†Noga Sikron-Persi[1], Gideon Grafi[1]*

[1]French Associates Institute for Agriculture and Biotechnology of Drylands, Jacob Blaustein Institutes for Desert Research, Ben-Gurion University of the Negev, Midreshet Ben Gurion 84990, Israel. [2]Institute of Plant Science and Resources, Okayama University, Kurashiki, Okayama, 710-0046, Japan.

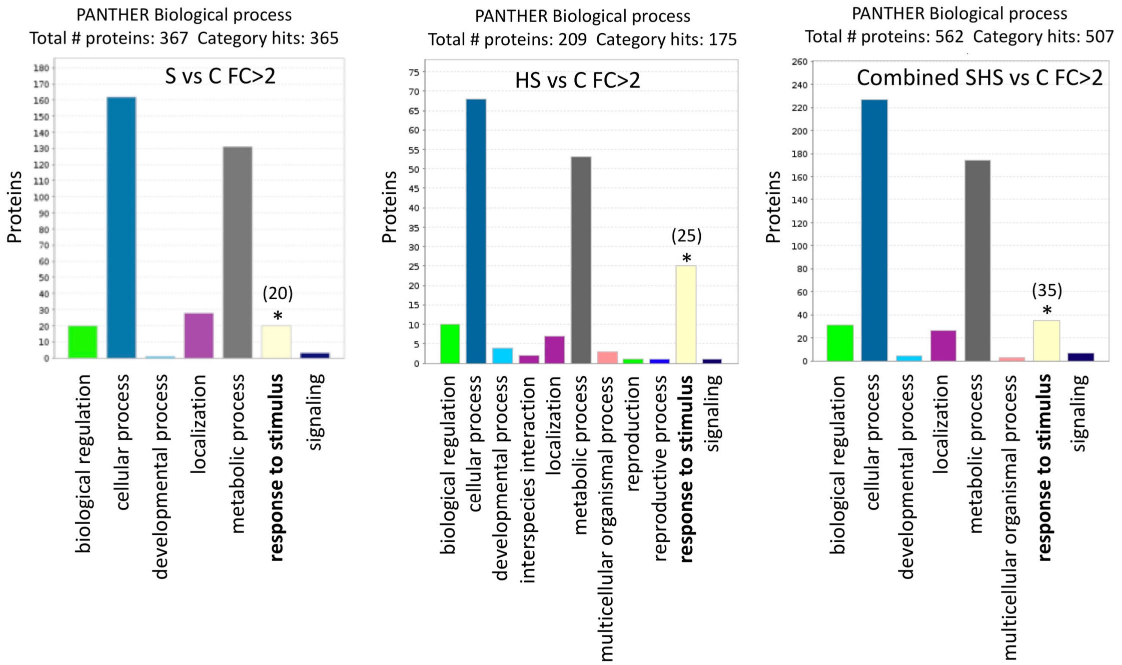

Fig. S1. Biological process categorization (PANTHER v.16) of differentially present (DP) proteins in dead pericarps derived from stress-treated plants (salt, S; Heat shock, HS; S+HS, SHS) vs. control. The category related to response to stimulus is marked by asterisk and the number of DP proteins in this category is given in brackets.

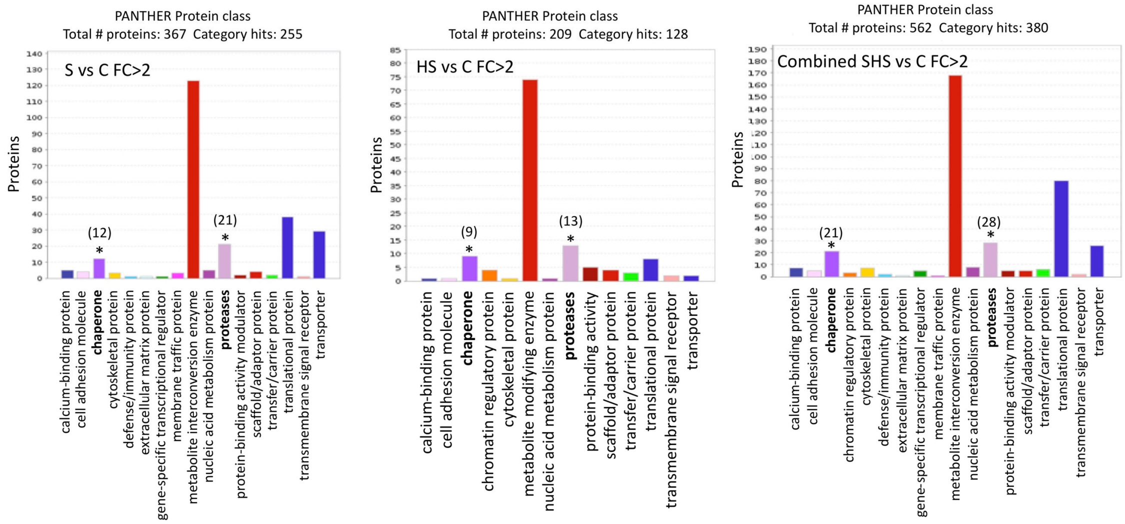

Fig. S2. Protein class categorization (PANTHER v.16) of DP proteins up-accumulated in dead pericarps of stress-treated plants vs. control. The chaperone and protease categories are marked by asterisks and the number of DP proteins is given in brackets.

Reference:

Huaiyu Mi, Dustin Ebert, Anushya Muruganujan, Caitlin Mills, Laurent-Philippe Albou, Tremayne Mushayamaha, Paul D Thomas, PANTHER version 16: a revised family classification, tree-based classification tool, enhancer regions and extensive API, *Nucleic Acids Research*, Volume 49, Issue D1, 8 January 2021, Pages D394–D403,
